# The Cost Effectiveness of Genomic Medicine in Cancer Control: A Systematic Literature Review

**DOI:** 10.1007/s40258-025-00949-w

**Published:** 2025-03-29

**Authors:** Mackenzie Bourke, Aideen McInerney-Leo, Julia Steinberg, Tiffany Boughtwood, Vivienne Milch, Anna Laura Ross, Elena Ambrosino, Kim Dalziel, Fanny Franchini, Li Huang, Riccarda Peters, Francisco Santos Gonzalez, Ilias Goranitis

**Affiliations:** 1 Economics of Genomics and Precision Medicine Unit, Centre for Health Policy, Melbourne School of Population and Global Health, University of Melbourne, 207 Bouverie Street, Melbourne, VIC 3053 Australia; 2 Frazer Institute, Dermatology Research Centre, The University of Queensland, Brisbane, QLD Australia; 3 The Daffodil Centre, The University of Sydney, a joint venture with Cancer Council NSW, Sydney, NSW Australia; 4 Australian Genomics, Murdoch Children’s Research Institute, Melbourne, VIC Australia; 5 Cancer Australia, Sydney, NSW Australia; 6 Caring Futures Institute, Flinders University, Adelaide, SA Australia; 7 Science Division, World Health Organization, Geneva, Switzerland; 8 Child Health Economics Unit, School of Population and Global Health, Centre for Health Policy, University of Melbourne, MelbourneMelbourne, VIC Australia; 9 Faculty of Medicine, Dentistry and Health Sciences, Cancer Health Services Research, Centre for Health Policy, Melbourne School of Population and Global Health, The University of Melbourne, Melbourne, VIC Australia; 10 Department of Cancer Research, Peter MacCallum Cancer Centre, Melbourne, VIC Australia

## Abstract

**Background and Objective:**

Genomic medicine offers an unprecedented opportunity to improve cancer outcomes through prevention, early detection and precision therapy. Health policy makers worldwide are developing strategies to embed genomic medicine in routine cancer care. Successful translation of genomic medicine, however, remains slow. This systematic review aims to identify and synthesise published evidence on the cost effectiveness of genomic medicine in cancer control. The insights could support efforts to accelerate access to cost-effective applications of human genomics.

**Methods:**

The study protocol was registered with PROSPERO (CRD42024480842), and the review was conducted in line with Preferred Reporting Items for Systematic Reviews and Meta Analyses (PRISMA) Guidelines. The search was run in four databases: MEDLINE, Embase, CINAHL and EconLit. Full economic evaluations of genomic technologies at any stage of cancer care, and published after 2018 and in English, were included for data extraction.

**Results:**

The review identified 137 articles that met the inclusion criteria. Most economic evaluations focused on the prevention and early detection stage (*n* = 44; 32%), the treatment stage (*n* = 36; 26%), and managing relapsed, refractory or progressive disease (*n* = 51, 37%). Convergent cost-effectiveness evidence was identified for the prevention and early detection of breast and ovarian cancer, and for colorectal and endometrial cancers. For cancer treatment, the use of genomic testing for guiding therapy was highly likely to be cost effective for breast and blood cancers. Studies reported that genomic medicine was cost effective for advanced and metastatic non-small cell lung cancer. There was insufficient or mixed evidence regarding the cost effectiveness of genomic medicine in the management of other cancers.

**Conclusions:**

This review mapped out the cost-effectiveness evidence of genomic medicine across the cancer care continuum. Gaps in the literature mean that potentially cost-effective uses of genomic medicine in cancer control, for example rare cancers or cancers of unknown primary, may be being overlooked. Evidence on the value of information and budget impact are critical, and advancements in methods to include distributional effects, system capacity and consumer preferences will be valuable. Expanding the current cost-effectiveness evidence base is essential to enable the sustainable and equitable translation of genomic medicine.

**Supplementary Information:**

The online version contains supplementary material available at 10.1007/s40258-025-00949-w.

## Key Points for Decision Makers

**Table:** 

Genomic medicine was likely to be cost effective for the prevention and early detection of breast and ovarian cancer, and colorectal and endometrial cancers (Lynch syndrome).
For cancer treatment, the use of genomic testing for guiding therapy was highly likely to be cost effective for breast and blood cancers and there was limited evidence suggesting that genomic medicine may not be cost effective in the treatment of colorectal cancers.
The use of genomic medicine in managing refractory, relapsed or progressive cancer, and end of life care, was likely to be cost effective in the management of advanced and metastatic non-small cell lung cancer.
For most cancers at most stages of care, and for low- and middle-income countries, there is insufficient evidence to form conclusions on the likelihood of cost effectiveness. To inform decision making, the cost-effectiveness evidence base should be expanded to capture all potentially high-value applications of genomic medicine in cancer control.

## Introduction

Cancer is a leading cause of mortality globally, with an estimated 10 million deaths annually around the world, 65% of which take place in low- and middle-income countries [1, 2]. Cancer mortality is expected to increase to 35 million by 2050 [3]. Genetic variations in human genomes, such as hereditary or environmental factors, determine cancer susceptibility and development and may influence therapeutic outcomes. Genomic medicine (GM) uses genomic information to understand the risk of developing cancer and enable targeted treatment decisions [4, 5], providing an unprecedented opportunity in cancer control through prevention, early detection and precision therapy [6, 7]. Governments worldwide are developing strategies to embed genomics across the cancer care continuum [8, 9], and the World Health Organization has acknowledged the critical role of GM in improving population health and provided recommendations to accelerate access [10].

Despite rapid scientific developments [11], successful translation of GM into healthcare systems has been slow. Progress has been limited by health infrastructure and resources, such as workforce and laboratory capacity, availability and procurement of genomic technologies [12, 13], and limited economics evidence on the likely sustainability and cost effectiveness of these technologies [14, 15]. Health economics evidence is critical for informing changes in practices and accelerating access to human genomics [16]. To support policy development and inform prioritisation decisions [15, 17], a deeper understanding of the cost effectiveness of GM in cancer control by cancer type and care stage is essential for a sustainable and equitable translation of GM into clinical practice and population health.

Previous reviews in this space have either focused on selected cancer types (e.g. familial breast and ovarian cancer) or particular stages of cancer management [18–20]. This systematic literature review broadens the scope by identifying and synthesising the evidence on cost effectiveness of GM across various cancer types and stages of the cancer care continuum. The review provides insights into the current evidence of the cost effectiveness of GM in cancer control and may provide useful insights to inform the development of national and international frameworks for genomics investment and translation, or support health economic evaluations in this space.

## Methods

The study protocol was registered with PROSPERO (CRD42024480842), and review was conducted in line with Preferred Reporting Items for Systematic reviews and Meta-analyses (PRISMA) Guidelines [21].

### Search Strategy

The search strategy was developed in collaboration with a biosciences research librarian at the University of Melbourne. The search terms were collated through examination of reviews related to GM and cancer [19, 22–29] (Table 1 of the Electronic Supplementary Material [ESM]), combined with health economics keywords including some used in prior reviews [19, 22, 25, 28, 29]. The search strategy was applied across four databases: MEDLINE and Embase via the Ovid platform, Cumulated Index to Nursing and Allied Health (CINAHL), and EconLit via the EBSCOhost platform. The final search used medical subject headings and free text and was limited to title and abstract. The search terms for free text were combined using Boolean operators. Because of the rapid development in the field of GM and based on clinical expert advice on the balance between relevance and quantity of evidence, search results were limited to human studies published in English language between 1 January, 2018 and 12 December, 2023.

### Study Selection

All search results were downloaded and imported into Covidence systematic review software (Veritas Health Innovation). Duplicates were removed and three reviewers were responsible for title and abstract screening. Articles were included if they were full economic evaluations of genomic technologies at any stage of cancer care (inclusion/exclusion criteria detailed in Table 2 of the ESM). A single reviewer (MB) conducted all title and abstract screening, with a random subset of 20% of articles being reviewed by a second reviewer (RP or FSG) to ensure consistency and low discrepancy. Discrepancies were managed by group discussion with senior investigator (IG). Full-text articles were citation tracked to identify additional relevant research articles.

### Data Extraction

A data extraction template was developed using Covidence systematic review software. As for the study selection phase, a single reviewer (MB) conducted all data extraction, with a random subset of 20% of articles being reviewed by a second reviewer (either RP or FSG). A 20% random sample for independent double review is consistent with PRISMA guidelines and has precedence in literature [30, 31]. Data were extracted that were relevant to the study context in terms of cancer care and genomic technology, the economic evaluation methods and conclusions of the research. Study context included the type of cancer being considered, the stage on the care continuum, the type of genomic technology intervention, comparator and the country. Methodological components included the type of the economic evaluation, perspective, cost components, cost sources, outcomes, model type, model structure, utility sources, currency, costing year and sensitivity analyses. Further, information on results were extracted, such as the estimated costs and outcomes, incremental cost-effectiveness ratio (ICER), threshold applied, and probability of being cost effective or dominant. In economic evaluations, ‘dominance’ means that a strategy is cheaper and more effective. ‘Cost-effective’ means that a strategy costs more and offers better outcomes, and considered good value for money in the context of a given value judgement threshold [32]. Information on conflict of interest (COI) was further extracted. More specifically, we extracted information on whether (a) COI is acknowledged but no conflicts are reported; (b) COI is reported but the manuscript is academic-led research (i.e., lead or corresponding authors are affiliated with a university; (c) COI is reported but the manuscript is industry-led research; and (d) COI statement is not provided at all. A full description of the data extracted is included in Table 3 of the ESM.

### Data Analysis

Economic evaluation evidence was organised into the stages on the cancer continuum: prevention and early detection, treatment, managing refractory, relapsed or progressive disease, and palliative and end-of-life care. Results were further stratified by cancer type. As the contexts were heterogenous, no meta-analysis was undertaken and results reported were narratively reviewed and synthesised. To allow for a comparison of the incremental cost-effectiveness, results from each economic evaluation were tabulated and compared to the threshold applied in the study. All costs were converted to USD based on historical conversion rates [33] for the 31 December in the costing reference year of the study, and are presented below in USD unless otherwise specified.

## Results

**Fig. 1:**
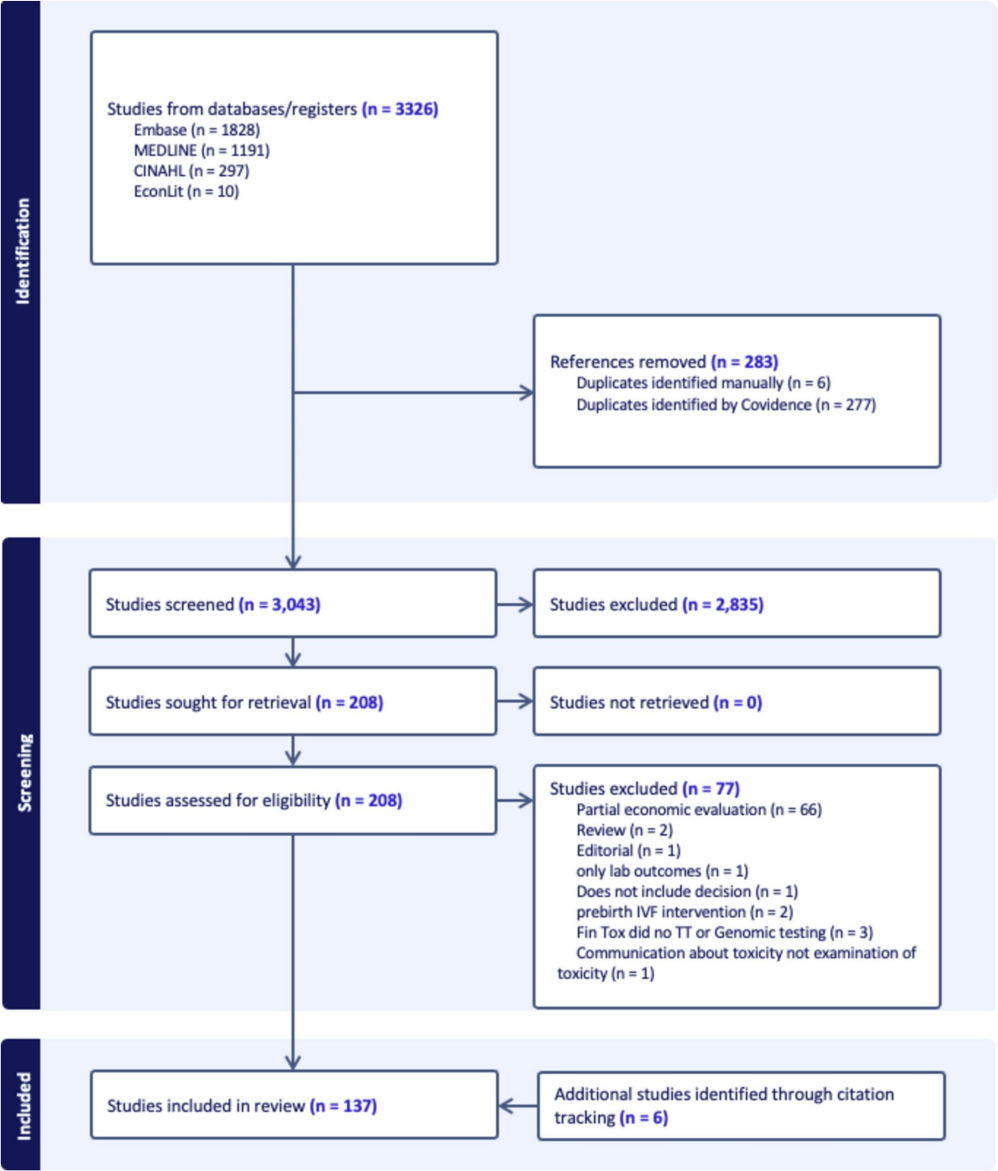
Preferred Reporting Items for Systematic Reviews and Meta Analyses (PRISMA) diagram. *Fin Tox* financial toxicity, *IVF* in vitro fertilisation, *TT* targeted therapies

The search results are outlined in Fig. 1. The initial search yielded 3326 articles. After removing 283 (9%) duplicates, 2785 (92%) articles were excluded following a screening of titles and abstracts, with a further 77 (3%) articles excluded following a full-text review (see Fig. 1 PRISMA diagram). An additional five articles were identified and added through citation tracking of the included articles. In total, 137 articles were included for data extraction.

A summary of the cost-effectiveness evidence is presented in Table 1. The use of GM in the prevention and early detection of cancer made up a substantial share of the evaluations identified in the review (*n* = 44; 32%). In this stage of cancer care, 80% of economic evaluations focused on either breast and ovarian cancer (*n* = 21) or colorectal and endometrial cancer (*n* = 14). Genomic medicine in the treatment stage of cancer care included 36 economic evaluations (26%), mainly for breast (*n* = 12), blood (*n* = 8), and colorectal cancers [CRC] (*n* = 6). There were 51 (37%) economic evaluations that considered the cost effectiveness of GM for relapsed, refractory or progressive cancer exclusively, of which 45% were for lung cancer. Studies were predominantly (72%) conducted in either Europe (59 evaluations) or North America (55 evaluations), with 86% of them focusing on high-income countries (Table 5 and Fig. 1 of the ESM) and 98% on adult patients.

**Table 1:** Cost effectiveness of genomic medicine across the care continuum

Continuum point	Cancer type	Count	Dominant	Cost effective	Uncertain	Not cost effective	% Cost effective
Prevention and early detection (*n* = 44; 32%)	Breast and ovarian	21	3	18	0	0	100%
	Colorectal and endometrial	14	1	11	1	1	86%
	Multi-cancer	5	2	2	0	1	80%
	Prostate	4	0	2	2	0	50%
Diagnosis staging and planning (*n* = 4; 3%)	Breast and ovarian	1	0	1	0	0	100%
	Colorectal and endometrial	1	0	0	1	0	0%
	Thyroid	1	0	0	0	1	0%
	Melanoma	1	1	0	0	0	100%
Treatment (*n* = 36; 26%)	Breast	12	5	4	3	0	75%
	Blood cancers	8	3	4	1	0	88%
	Colorectal	6	0	2	4	0	33%
	Gastrointestinal cancers	2	0	1	1	0	50%
	Gynaecological cancers	2	0	2	0	0	100%
	Brain and central nervous system cancers	2	1	0	1	0	50%
	Endocrine cancers	1	0	1	0	0	100%
	Tumour agnostic	3	0	0	1	2	0%
Refractory, relapsed or progressive (*n* = 51; 37%)	Lung	23	2	12	4	5	61%
	Breast cancer	3	1	1	0	1	67%
	RCC	6	0	4	2	0	67%
	Melanoma	10	0	3	2	5	30%
	Cholangiocarcinoma	2	0	0	0	2	0%
	Leukaemia	1	0	1	0	0	100%
	Prostate	3	0	1	1	1	33%
	Gynaecological cancers	1	0	0	0	1	0%
	Gastrointestinal cancers	1	0	1	0		100%
	Tumour agnostic	1	0	0	0	1	0%
End-of-life care (*n* = 2; 1%)	Multi-cancer	1	0	0	0	1	0%
	RCC	1	0	1	0	0	100%

*RCC* renal cell carcinoma

### Prevention and Early Detection (*N* = 44)

Breast and ovarian cancer, including hereditary breast and ovarian cancer. The review identified 44 (32%) cost-effectiveness studies that focused on prevention and early detection of cancer, with 48% of studies being on breast and ovarian cancer. Two prevention and early detection strategies were generally applied: (A) universal population screening, whereby an entire population received genomic screening at a certain age; or (B) proband-based screening, whereby patients newly diagnosed with breast or ovarian cancer were tested, and those who were positive for specific variants had their first-degree (and possibly second-degree) relatives screened. In both strategies, those with a high cancer risk had preventative strategies offered to them or were given access to additional regular cancer screening, such as mammography.

Population-based approaches were reported to be either more effective and less costly (i.e. dominant) [34, 35] or cost effective [36–45] compared to no or standard cancer screening approaches across all 12 studies identified. The studies primarily focused on rare variants that strongly increased cancer risk; however, two articles focused on an alternate emerging genetic test, polygenic risk scores (PRS). Dominance of PRS was demonstrated in Singapore by Wong and Chai, who showed that PRS based breast cancer screening improved outcomes and reduced costs by $2722 (SGD 3671) per person tested compared to a standard screening approach [34]. Pashayan et al. demonstrated that compared with age-based screening in the UK, PRS-based screening from the 32nd percentile of risk and above increased quality-adjusted life-years (QALYs) by 450 and decreased costs by $25,605 (£20,066) in a cohort of 10,000 women [35]. For the remaining evaluations, cost effectiveness was demonstrated with a highest ICER of $92,600 per QALY gained in the USA [36]. Although all population-based screening studies identified here were either cost saving or cost effective, Manchanda et al. claimed that population-based screening was cost effective in high- (UK, USA and the Netherlands) and middle (China and Brazil) income countries compared to family history-based screening, but not cost effective in low-income (India) countries at $31,217 because of a lower threshold being applied [40].

Proband-based genomic screening was demonstrated to be dominant [46] or cost effective [47–54] in all nine articles identified [47–54]. Cost-effectiveness estimates ranged from cost saving in Canada (ICER − $6537) [46] to $37,940 (€31,621) per QALY gained in Spain. Of the nine articles, three considered probands with breast cancer [51, 52, 54], three considered probands with ovarian cancer [46, 47, 49], and three considered probands with either breast or ovarian cancer [48, 50, 53].

#### Colorectal and Endometrial Cancer, Including Lynch Syndrome

The review identified 14 articles (32%) that considered the cost effectiveness of screening strategies for the prevention and early detection of CRC, endometrial cancer or a combination of both. Of these, half evaluated population-based approaches and half proband-based approaches. Population-based testing was demonstrated to be dominant [55] or cost effective [55–59] in five out of seven of the articles identified. The study by Pereira et al. [60] reported that population-based genetic polymorphism testing in Portuguese adults aged over 40 years compared to no testing was unlikely to be cost effective (based on an ICER of $50,812 and a threshold of $50,000). This study only considered genetic variants in a particular biological pathway (the COX-2/PGE_2_ pathway), noting future research should consider testing wider sets of genes [60]. The study by Naber et al. found that compared to no screening, a PRS-stratified colonoscopy was cost effective; however, it was not cost effective compared to uniform screening in USA adults. A scenario analysis demonstrated that PRS became cost effective at a cost of $100 or when discriminatory performance of PRS was improved (at area under the curve = 0.65) [61].

Proband-based testing for Lynch syndrome was found to be cost effective in all seven articles compared with no testing [62–68]. However, several evaluations utilised a stepwise approach where immunohistochemistry and methylation were used prior to genomic sequencing, making the most cost-effective sequence of testing less clear. For example Stinton et al. demonstrated that while upfront germline testing for all patients diagnosed with CRC was cost effective compared with no testing, it was less effective and more costly than a stepwise approach that utilised immunohistochemistry followed by methylation and then germline testing [67]. Similarly, Snowsill et al. demonstrated that a stepwise process that used four-MMR protein immunohistochemistry followed by MLH1 testing, and then next-generation sequencing (NGS) was more cost effective than NGS for all probands when both were compared with a ‘no testing’ scenario in the UK [66].

#### Prostate Cancer

The cost -effectiveness of GM for prevention and detection of prostate cancer was evaluated in four articles (9%). Genomic screening was found to be cost effective in two of the articles [69, 70]. Both articles considered the use of the Sotckholm3 test, which uses genetic markers, blood protein markers and some clinical variables, to establish the patient risk profile, compared to standard screening using PSA and no screening. The remaining two articles showed borderline cost effectiveness. Callender et al. found that compared with no screening, PRS-based screening had a 48–57% probability of being cost effective between the willingness-to-pay thresholds of $26,000 (£20,000) and $39,386 (£30,000) per QALY gained [71]. Hendrix et al. considered different screening strategies to stratify surveillance intensity from a USA payer perspective and reported that the cost effectiveness of risk-stratified screening was uncertain and depended on the comparator being considered. For example, it reported that risk-stratified screening was cost effective compared with the standard biennial screening starting at 55 years, but not compared with a biennial screening schedule starting at 45 years [72].

#### Multi-cancer Testing

The review identified five articles (11%) that analysed the cost effectiveness of genomic testing for multiple cancers (where multiple cancers was defined as three or more), four pertained to adult populations and one pertained to paediatric populations. Taffazoli et al. demonstrated that multi-cancer early detection decreased costs and improved QALYs compared with routine testing over a lifetime horizon in the USA [73]. Similarly, Hackshaw et al. showed that multi-cancer early detection was cost saving per case detected in the UK and required an additional cost of $7060 per case detected in the USA compared with routine testing from a healthcare system perspective [74].

In Australia, Davidson et al. evaluated upfront genomic screening for several cancer susceptibility variants compared to targeted gene or gene panel testing for patients referred to a Familial Cancer Centre and showed that genomic screening led to an additional cost of $6740 (AUD $8744) per actionable variant detected [75]. Lacaze et al. considered genomic testing for three high-risk conditions in Australia (hereditary breast and ovarian cancer, Lynch syndrome and familial hypercholesterolaemia) and reported that at a per-test cost of $136 (AUD $200), genomic testing was cost effective compared to no testing at a threshold of $34,066 (AUD $50,000), with an ICER of $16,326 (ADU $23,963) per QALY gained [76].

Yeh et al. investigated the impact of universal newborn genetic screening using targeted NGS for paediatric cancer predisposition syndrome in the USA. The simulation model predicted a 7.8% decrease in cancer death by the age of 20 years for those in the targeted NGS group; however, the model also estimated that the incremental cost per additional life-year (LY) gained was $244,860, reporting that the intervention is unlikely to be cost effective over a lifetime horizon [77].

### Diagnosis, Staging and Planning (*N* = 4)

Diagnosis, staging and planning was the exclusive focus of four evaluations. Lim et al. (2018) reported that compared with routine clinical surveillance, BRCA mutation testing was cost effective in women with early-stage breast cancer in Malaysia when those testing positive were subsequently offered risk-reducing surgery ($2725 per additional QALY gained) [78]. Hao et al. evaluated the cost per Lynch syndrome case identified for eight different testing strategies; however, an incremental analysis was limited and therefore it is unclear how these strategies compared with current standard care [79]. A single evaluation demonstrated that genomic testing was dominated by surgical resection in thyroid cancer [80]. Another demonstrated that molecular testing dominated standard care in the diagnosis of melanoma in the USA [81].

### Treatment (*N* = 36)

Analyses focused on the cost effectiveness of treatment (*n* = 36; 26%) were generally conducted using one of two types of evaluation: (a) those that included testing to specify the risk or appropriateness of medication where some patients received standard therapy and others received targeted therapies and (b) those assuming genetic testing had already taken place and investigated the cost effectiveness of different targeted medications for a specific population.

#### Breast Cancer

The application of GM in the treatment of breast cancer was the focus of 12 (33%) cost-effectiveness analyses. Genomic testing was reported to be dominant [82–86] or cost effective [87–90] in nine of the evaluations. In the three remaining analyses, cost effectiveness was uncertain. Trentham-Dietz et al. considered a hypothetical test that could capture with 100% accuracy the prognosis for progression of ductal-carcinoma in situ [91]. They claimed that this test would dominate standard care in the USA. Wang et al. [92] showed that in the USA, using a 21-gene assay to direct chemotherapy decisions had an ICER of $62,200 per QALY gained, which would be considered cost effective against a $100,000 WTP threshold; however, they argued that the cost effectiveness was driven by the high-risk group, and the probability of cost effectiveness in the low-risk, intermediate-risk and high-risk groups was 18.4%, 55.1% and 96.6%, respectively. They further argued that most patients would be in the low-risk group in clinical practice and therefore, cost effectiveness is uncertain. Finally, in an evaluation by De Jongh et al., testing-guided treatment led to an estimated cost saving of $29,939,451 (€26,667,347) to the Dutch healthcare system, and 1364 fewer adverse events [93]. As adverse events were the only outcomes captured, the cost effectiveness was uncertain. Studies by Ibarrondo et al. and Perez Ramirez et al. presented findings from both the healthcare and societal perspective and demonstrated that when the perspective was widened to societal, the cost effectiveness of GM improved, largely driven by reduced chemotherapy related absenteeism [83, 87].

#### Blood Cancers

There were eight (22%) articles that considered the cost effectiveness of the treatment of blood cancers, with five studies focused on leukaemia, two on lymphoma and one on multiple myeloma. Genomic medicine in the management of leukaemia in adults was the focus of four evaluations comparing different combinations of targeted therapy, immunotherapy, and chemotherapy. Alrawashdh et al. compared the cost effectiveness of nine first-line therapies in the USA to a base case of venetoclax plus obinutuzumab (targeted therapy) from a healthcare system perspective over 10 years. It was reported that the base-case targeted therapy (venetoclax plus obinutuzumab) dominated the four chemoimmunotherapy agents considered, while four other targeted therapies that were considered improved the clinical outcome but were not cost effective, with ICERs that ranged from $501,236 to $869,300 per QALY gained for acalabrutinib plus obinutuzumab and ibrutinib plus rituximab, respectively [94]. Furthermore, Chatterjee et al. and Slot et al. reported that venetoclax plus obinutuzumab was cost effective in Canada and Denmark, respectively, from a healthcare system perspective [95, 96]. Finally, Munir et al. considered a comparison of two targeted therapy regimens and claimed that acalabrutinib monotherapy was cost effective compared to chlorambucil plus obinutuzumab in the USA with an ICER of $81,960 per QALY gained from a payer perspective, with 59% and 73% probability of cost effectiveness at a $100,000 and $150,000 WTP decision-making thresholds, respectively [97].

Only one evaluation of leukaemia treatment considered GM to guide treatment in children. Wei et al. used a decision tree for children in China with acute lymphoblastic leukaemia, claiming that NUD15 genetic testing-guided 6-mercaptopurine dosing dominated standard dosing over a 2-year time horizon from the perspective of the healthcare system [98].

Evaluations by Chen et al. and Regier et al. considered the cost effectiveness of molecularly guided treatment to manage diffuse large B-cell lymphoma from the USA and Canadian context, respectively, compared to standard R-CHOP treatment for all [99, 100]. Chen et al. reported that guided therapy led to an ICER of $15,015 per QALY gained and was cost effective at a WTP threshold of $50,000 per QALY [99]. Regier et al. (2022) found similar benefits however, had a lower probability of being cost effective in the Canadian context with an ICER of $57,024 (CAD $77,806) per patient and a 24.3% and 53.7% probability of being cost effective at $36,645 (CAD $50,000) and $73,290 (CAD $100,000) per QALY threshold, respectively [100]. Furthermore, Regier et al., considered molecularly guided therapy to improve second-line therapy outcomes, at which point it was reported to be more cost effective with an ICER of $38,777 (CAD $52,909) per QALY gained [100].

Multiple myeloma was the focus of a single evaluation by Gaultney et al. [101] in which FISH and SKY92 testing was used to stratify risk and guide treatment. Risk stratification guided treatment was claimed to dominate no testing in the Netherlands, Germany, the UK, France and Spain from a healthcare system perspective over a lifetime horizon [101].

#### Colorectal Cancer

The treatment of CRC was the focus of six evaluations identified. Of these, two found molecular testing-guided treatment cost effective. Chaudhari and Issa considered a 12-gene, 18-gene or 482-gene assay as well as an immunoscore assay followed by adjuvant chemotherapy for high-risk patients or no chemotherapy for low-risk patients in the USA. They found that the 12-gene immunoscore assay was the most cost-effective strategy with an ICER of $6037 per QALY gained compared with a no testing scenario. [102] Fragoulakis et al. compared genotyping-guided use of capecitabine, 5-fluorouracil and irinotecan with capecitabine, 5-fluorouracil to irinotecan without genotyping in Italy and estimated the ICER to be $16,408 (€13,418) per QALY gained, which they considered cost effective against a WTP threshold of $61,141 (€50,000) [103].

A further two evaluations reported that identifying molecular targets could improve the cost effectiveness of chemotherapy plus anti-epidermal growth factor receptor (EGFR) monoclonal antibody treatment; however, these evaluations did not consider a treatment decision using molecular testing and therefore the cost-effectiveness results are difficult to interpret. Harty et al. reported that cetuximab plus FOLFIRI compared with FOLFIRI alone was substantially more cost effective in patients with RAS mutation wild-type than in a standard CRC cohort, with ICERs of $56,381 and $167,068 per QALY, respectively [104]. Similarly, Lee et al. reported that in Hong Kong, chemotherapy plus an anti-EGFR monoclonal antibody compared to chemotherapy plus bevacizumab had an ICER of $76,537 and $106,847 per QALY in the pan-RAS wild-type left-sided tumour group and KRAS wild-type groups, respectively [105].

Jang et al. considered genomically guided surgery for patients with different biomarker profiles. It claimed that for patients with low-risk CRC, endoscopic surgery was the dominant strategy, while for patients with the highest-risk variants, laparoscopic surgery was most effective; however, it was not cost effective (ICER up to $178,765 per QALY gained) [106].

Finally, Kacew et al. evaluated the use of artificial intelligence in combination with NGS. Next-generation sequencing alone was used as the reference case and was reported to be the most effective, with an additional 3% of patients receiving appropriate targeted therapy, compared with artificial intelligence risk prediction plus NGS, but at an increased total health system cost of $10 million in the USA [107].

#### Gastrointestinal Cancers

Gastrointestinal cancers were the focus of two (6%) of the evaluations identified in the review Liu et al. conducted by a network meta-analysis and cost-effectiveness analysis of first-line immunotherapy and targeted therapy options for patients with unresectable hepatocellular carcinoma from a payer perspective in China. It was claimed that sintilimab plus a bevacizumab biosimilar had the greatest impact on overall survival, and camrelizumab plus rivoceranib increased progression-free survival and both fell under a WTP threshold of $37,653 per QALY gained [108]. Krepline et al. reported that germline testing to guide treatment compared to selective testing based on a family history in USA patients with pancreatic cancer was unlikely to be cost effective using a WTP threshold of $100,000 per LY gained, reporting an ICER of $121,942 per LY gained. However, it was reported that a selective testing strategy was more cost effective and had a 59% probability of cost effectiveness from a USA healthcare system perspective [109].

#### Gynaecological Cancers

An analysis by Liu et al. reported using a Markov model over a 7-year time horizon that combination immunotherapy plus targeted therapy (pembrolizumab plus lenvatinib) compared to chemotherapy (doxorubicin) in first-line therapy of USA patients with mismatch repair-proficient advanced endometrial cancer was cost effective at a threshold of $150,000 with an ICER of $110,401 per QALY gained from a USA payer perspective [110]. Orellana et al. compared the cost effectiveness of tumour molecular testing versus no testing and four other different test and treat scenarios, for USA patients with stage III endometrial cancer. Tumour molecular testing was reported to be cost saving compared with no tumour molecular testing scenario; however, it was claimed not cost effective compared to mismatch repair immunohistochemistry alone, at a WTP threshold of $100,000 per QALY with an ICER of $182,797 per QALY gained [111].

#### Brain and Central Nervous System Cancers

An evaluation by Rios et al. reported that compared to a no testing scenario, molecular testing to determine the *BRAF* mutation status of paediatric patients with low-grade glioma in Canada was dominant from a healthcare system perspective [112]. Ranjan et al. evaluated the costs and outcomes of cancer stem cell assay-guided chemotherapy in USA patients with unmethylated O6-methylguanine-DNA methyltransferase-promoter recurrent glioblastoma within a trial. It was reported that when patients received test-guided therapy they had an average LY gain of 0.275 and an additional cost $41,496 from a health system perspective (ICER $150,895 per LY gained, conducted in this systematic review) [113].

#### Endocrine Cancers

Tessler et al. reported, using a Markov model over a lifetime horizon, that molecular testing guided preventative surgery for USA patients with low-risk differentiated thyroid cancer, compared with a standard of care hemi-thyroidectomy based on clinical criteria led to an increase of 1.7 QALYs per patient at an incremental cost of $327, with an ICER of $190 per QALY gained, which was claimed to be cost effective at a threshold of $50,000 per QALY from a USA health system perspective [114].

#### Tumour Agnostic Treatments

The cost-effectiveness of GM in a tumour agnostic context was evaluated in three (8%) articles. Modelling approaches were applied in two of the evaluations and the other was conducted within a trial. The outcomes used included QALYs (*n* = 2) and LYs (*n* = 1), and the perspectives taken included health system/payer (*n* = 1), societal (*n* = 1) or both (*n* = 1).

Huygens et al. considered neurotrophic tyrosine receptor kinase testing. Tumour agnostic neurotrophic tyrosine receptor kinase testing was reported to not be cost effective from a societal perspective in the Netherlands [115].

Fragoulakis et al. considered the cost effectiveness of cancer treated with fluoropyrimidines in those with and without dihydropyrimidine dehydrogenase gene mutations in Italy. The analysis reported that health outcomes were better and costs were lower in those without a dihydropyrimidine dehydrogenase gene mutation [116]. Finally, Weymann et al. reported that participants who have had comprehensive genomic sequencing in Canada had higher hospital costs of $3757 (CAD $5203) and no significant difference in outcomes [117].

### Managing Refractory, Relapsed or Progressive Disease (*N* = 51)

Evaluations focusing on refractory, relapsed or progressive disease made up the largest portion of the review (*n* = 51; 37%).

#### Lung Cancer

There were 23 (45%) economic evaluations of GM for advanced or metastatic non-small cell lung cancer identified. These articles constituted the largest portion of articles in the refractory, relapsed or progressive disease stage of cancer care.

The use of specific biomarker testing to guide treatment was the focus of six of the articles [118–123], with three claimed that single gene testing to target therapy for anaplastic lymphoma kinase or *EGFR* was cost effective compared to standard chemotherapy for all patients [118–120]. The addition of proto-oncogene tyrosine-protein kinase ROS (*ROS1*) testing to standard-of-care sequential testing was also claimed to be cost effective [121]. Lu et al. reported that anaplastic lymphoma kinase testing was cost effective compared with standard-of-care chemotherapy for patients in the Patient Assistance Program in China (a programme that provides financial assistance to access medicine for patients with low-income and chronic disease); however, it was reported not cost effective for patients who were not in the Patient Assistance Program [122]. An additional article reported that *EGFR* testing followed by targeted erlotinib, gefitinib or afatanib had borderline cost effectiveness compared with standard chemotherapy for all in Brazil [123].

Next-generation sequencing or multigene testing was considered in nine of the evaluations identified [124–132]. Multigene testing was reported to be cost effective in six out of seven evaluations identified when compared with sequential testing (testing genes individually until a positive result is returned). The remaining article by Zou et al. considered the cost effectiveness at the cohort level of 50% of patients receiving comprehensive genomic profiling of 324 genes, and 50% receiving NGS of 23 genes compared to five single gene strategies with a proportion of patients receiving each, it was estimated that the 50/50 comprehensive genomic profiling/NGS strategy cost $238,876 per QALY gained and was therefore not cost effective [130].

A further two articles considered both multigene and sequential genetic testing strategies compared to no testing. In the study by Hofmarcher et al., sequential testing was reported to be dominant compared to no testing in Brazil, Germany, Poland, Turkey, South Africa, China, Japan and Australia and cost effective in the USA. Multigene testing was reported to be dominant in Brazil, Poland, Turkey, South Africa and China, cost effective in Japan, Australia and Germany, but not cost effective in the USA because of large medication costs [131]. Loubiere et al. considered both the multigene panel of four genes and a single Kirsten Rat Sarcoma Virus (*KRAS*) panel compared to no testing in France, both were claimed to be highly likely to be cost effective [132]. The use of whole genome sequencing was reported to not be cost effective as either a first line [133, 134] or final test [133] for patients who received negative results on sequential testing compared to sequential biomarker testing alone [133, 134].

A liquid biopsy was considered in addition to a tissue biopsy in three of the evaluations identified, and the results were mixed. Ezeife et al. claimed that the addition of a liquid biopsy to a tissue biopsy dominated the tissue biopsy alone in Canada over a 2-year time horizon [135]. However, Englmeier et al. reported that in Germany the addition of a liquid biopsy had borderline cost effectiveness with an ICER of $65,920 (€53,909) per QALY gained [136]. Furthermore, Cho et al. found uncertainties in results using a number of treated sensitising mutations: while the cost was decreased at first-line treatment, it increased at second-line treatment [137]. Two articles focused specifically on adults with metastatic adenocarcinoma of NSCLC and reported that the use of genomic testing to guide targeted therapy was not cost effective, owing largely to the high costs of the targeted therapies [138, 139].

Leung et al. considered the use of immunotherapy agents in adult patients with NSCLC who had progressed on platinum-based chemotherapy. It was reported that pembrolizumab, nivolumab and atezolizumab were all cost effective in Taiwan for second-line treatment of NSCLC at a threshold of $74,203 (NTD 2,221,930) per QALY gained compared to the chemotherapy agent docatexel [140]. Respective ICERs for pembrolizumab, nivolumab and atezolizumab were $13,896 (NTD 416,102), $52,529 (NTD 1,572,912) and $52,781 (NTD 1,580,469) per QALY gained.

#### Breast Cancer

Three articles considered breast cancer. Saito et al. reported that *BRCA1/2* mutation profiling to guide olaparib treatment in Japanese patients with HER2-negative or triple-negative metastatic breast cancer who had previously undergone chemotherapy, compared to standard-of-care chemotherapy, was not cost effective at a threshold of $91,146 (JPY 10,000,000) with an ICER of $133,777 (JPY 14,677,259) per QALY gained [141]. Ren et al. reported that neratinib plus capecitabine compared to lapatinib plus capecitabine for the third-line management of Chinese patients with HER2 plus metastatic breast cancer was dominant in 83% of simulations [142]. Pennarun et al. reported that the use of a recurrence index for distant recurrence compared to a no testing scenario in women with an HER2-negative early-stage breast cancer in Taiwan was cost effective at a WTP threshold of $25,787 (NTD 790,000) per QALY gained, with an ICER of $5675 (NTD 173,842) per QALY gained [143].

#### Renal Cell Carcinoma

The review identified six evaluations that considered the use of GM and targeted therapies to treat advanced or metastatic RCC. Nazha et al. reported in a Canadian context that for the treatment of RCC, sunitinib was cost effective compared to pazopanib at a threshold of $79,524 (CAD $100,000) with an ICER of $53,469 (CAD $67,227) per QALY gained [144]. Zhu et al. reported that compared to sunitinib, first-line lenvatinib plus pembrolizumab had a 59% probability of being cost effective at a threshold of $150,000 with an ICER of 131,656 per QALY [145]. Chen et al. claimed that pharmacokinetically guided sunitinib compared to standard dose sunitinib was dominant in both a Chinese and USA context [146]. Redig et al. reported that since the introduction of targeted therapies for RCC in Sweden, cost effectiveness in practice has been improving, and that incremental cost effectiveness of targeted therapies compared to standard therapies decreased from $78,656 per LY gained between 2006 and 2009 to $78,656 per LY gained between 2009 and 2010 [147]. Chandler et al. [148] and Meng et al. [149] considered the cost effectiveness of cabozantinib versus everolimus, axitinib and nivolumab in subsequent-line RCC in Japan and England, respectively. In both evaluations, cabozantinib was reported to dominate nivolumab. However, in Japan it was reported to be cost effective compared to everorlimus and axitinib at a threshold of $57,251 per QALY gained (JPY 7.5M) with respective ICERs of $41,034 (JPY 5,375,559) and $16,970 per QALY gained [148]. In England cost effectiveness was considered to be borderline compared to axitinib at a threshold of $127,602 (£100,000) per QALY gained with an ICER of $126,284 (£98,967) per QALY gained, and not cost effective compared to everolimus with an ICER of $175,389 (£137,450) per QALY gained [149].

#### Melanoma

The review identified ten evaluations that considered the cost-effectiveness of GM in the treatment of advanced or metastatic melanoma. All ten articles compared immunotherapies and targeted therapies, eight of them referred specifically to v-raf murine sarcoma viral oncogene homolog B1 (*BRAF*)-mutated melanoma [150–157]. The remaining two evaluations considered high-risk advanced or metastatic melanoma that was not exclusively BRAF mutated [158, 159]. Wahler et al. and Mulder et al. compared immunotherapy and targeted therapy against routine surveillance in Germany and the Netherlands, respectively, and reported that both were cost effective [156, 159]. Furthermore, Charpentier et al. considered immunotherapies and targeted therapies together as ‘new therapies’ and reported them to be cost effective compared to chemotherapies in France at a WTP threshold of $122,281 (€100,000) per LY gained with an ICER of $110,278 (€90,184) [158]. However, this result is difficult to interpret as pre- and post-data were used, the model was inadequately described and the value judgement threshold applied is unconventional.

Targeted therapy and immunotherapies were directly compared in five of the evaluations identified. Wu et al. and Bensimon et al. reported that targeted therapy (dabrafenib plus trametinib) was dominated by pembrolizumab in the USA [150, 151]. In three of the articles, dabrafenib and trametinib was estimated to be more effective than immunotherapy; however, it was not deemed cost effective by standard reported thresholds [152–154]. In the evaluation by Tarhini et al., first-line treatment with anti-programmed cell death-1 (anti-PD1) inhibitors followed by second-line BRAF and MEK inhibitors was claimed to be cost effective, compared to first-line BRAF and MEK inhibitors followed by second-line anti-PD1 therapy, at a WTP threshold of $150,000 per QALY gained with a reported ICER of $79,124 per QALY gained [157].

The remaining evaluation considered treatment strategies that incorporated both immunotherapies and targeted therapies. Kandel et al. reported that mono-targeted therapy for first-line treatment followed by anti-PD1 therapy for second line dominated all other treatment strategies. The most cost-effective strategy for *BRAF*-mutated melanoma patients in France was a mono-targeted therapy for first-line treatment followed by anti-PD1 therapy for second line [155].

#### Cholangiocarcinoma

Cholangiocarcinoma was evaluated by two analyses identified in the review; both considered the cost effectiveness of pemigatinib as a second-line therapy. Chen et al. reported that in patients with advanced intrahepatic cholangiocarcinoma with fibroblast growth factor receptor 2 fusions in Taiwan, pemigatinib was not cost effective at $95,595 (NTD 2,928,570) compared to two comparators, combination oxaliplatin, L-folinic acid and fluorouracil with respective ICERs of $189,805 (NTD 5,814,700) and $175,624 (NTD 5,814,700) per QALY [160]. Chueh et al. [161] showed that pemigatinib monotherapy and triplet chemotherapy (mFOLFOX) regimens based on fibroblast growth factor receptor 2 status in patients with advanced intrahepatic cholangiocarcinoma in Taiwan was not cost effective compared to fluorouracil chemotherapy at a threshold of $104,264 (NTD 2,889,684) with an ICER of $123.077 (NTD 3,411,098) per QALY [161].

#### Leukaemia

An evaluation by Pandya et al. [162] reported gilternitib was cost effective compared to standard care as well as best supportive care in patients with relapsed/refractory FMS-like tyrosine kinase 3 acute myeloid leukaemia in the USA at a threshold of $150,000 with respective ICERs of $115,192 and $107,435 per QALY [162].

#### Prostate Cancer

The review identified three (6%) evaluations that considered the cost effectiveness of the management of advance or metastatic prostate cancer. Two of the trials considered the cost effectiveness of targeted therapy plus androgen deprivation therapy (ADT) to ADT alone, and the results were reported as mixed. Parmar et al. reported from a health system perspective that apalutamide plus ADT was not cost effective with an ICER of $129,135 per QALY (CAD $164,700), which exceeded the WTP threshold of $78,406 (CAD $100,000) over a lifetime horizon [163]. Barbier et al. claimed using a Markov model that ADT plus abiraterone was cost effective from a Swiss healthcare system perspective compared to ADT alone in patients with metastatic hormone-sensitive prostate cancer, with an ICER of $22,293 (€29,596) per QALY gained over a 30-year time horizon [164].

Su et al. used a partitioned-survival model to evaluate the cost effectiveness of olaparib from a USA payer perspective compared to standard of care for patients with metastatic castration-resistant prostate cancer when patients had the presence of one of three gene alterations (*BRCA1, BRCA2* or *ATM*) and when patients had the presence of 1 of 15 gene alterations (*BRCA1, BRCA2, ATM, BRIP1, BARD1, CDK12, CHEK1, CHEK2, FANCL, PALB2, PPP2R2A, RAD51B, RAD51C, RAD51D* or *RAD54L*). It was reported that for patients with 1 of three alterations, olaparib had an ICER of $116,903, and when patients had 1 of 15 alterations, olaparib dominated standard of care. [165] Unfortunately, this did not capture the costs for patients who tested negative and therefore interpreting cost effectiveness is difficult.

#### Gastrointestinal Cancers

Banerjee et al. reported, using a Markov model over a 10-year time horizon, that first-line imatinib followed by sunitinib compared to empirical imatinib in patients with metastatic gastrointestinal stromal tumours in the USA was cost effective at $100,00 per QALY, with an ICER of $92,100 per QALY gained from a US payer perspective [166].

#### Gynaecological Cancers

Richardson et al. reported, using a Markov model, that three potential treatment sequences of chemotherapy, immunotherapy and targeted therapy compared to standard of care chemotherapy and targeted therapy in USA patients with advanced metastatic and recurrent cervical cancer improved outcomes. However all three sequences showed a negative net monetary benefit compared to standard of care from a health system perspective when QALYs were valued at $150,000 [167].

#### Tumour Agnostic Treatments

Vellekoop et al. analysed four tumour agnostic neurotrophic tyrosine receptor kinase testing strategies in a target population of adults with locally advanced or metastatic solid tumours, who had previously received one or more lines of treatment. Neurotrophic tyrosine receptor kinase testing was reported to have a negative net-monetary benefit in England, Hungary and the Netherlands compared to a not testing scenario [168].

### Palliative Care and End of Life (*N* = 2)

The review only identified two (2%) evaluations that considered the use of genomic technologies for end-of-life care in patients with cancer. Ree et al. used a partitioned-survival model over a 10-year time horizon to evaluate molecularly targeted matched off-label therapies for multiple cancer types in Norway. The analysis was based on the results of the MetAction study, which compared GM to best supportive care, and an indirect comparison based on published randomised controlled trials in similar populations [169]. Compared with the controls from the RECOURSE and CORRECT trials, the ICER for molecularly targeted therapy was reported as not cost effective with respective ICERs of $154.395 (€126,262) and $134,012 (€109,593), which exceeded the WTP threshold of $68,953 (€56,389) per QALY gained [169]. Edwards et al. demonstrated, using a partitioned-survival model over a lifetime horizon, that compared to best supportive care everolimus was cost effective at a WTP threshold of $73,816 (£50,000) per QALY gained with an ICER of $66,436 (£45,000) per QALY gained compared to best supportive care for patients with previously treated RCC in the UK, the remaining treatments (axitinib, cabozantinib, everolimus, nivolumab and sunitinib) were reported as unlikely to be cost effective from a health system perspective [170]. Table 1 shows the cost effectiveness of GM across all aspects of the cancer care continuum for common cancer types.

### Conflict of Interest

Overall, economic evaluations reported COI well (Appendix 1 of the ESM). Only three articles (2%) did not report on COI [47, 80, 99]. Studies that reported no potential COI made up the largest portion of the articles. (42%) Evaluations that reported COI but were academic led made up 21% of the articles and articles that reported COI that were industry led made up 35% of the evaluations. The proportion of industry led articles was highest in the treatment stage where 45% of articles were reported to be industry led.

### Economic Evaluation Methods

The methods applied by the economic evaluations are outlined in Table 4 of the ESM. Across the continuum, most evaluations (98%) used modelling-based approaches, rather than evaluations alongside trials. Markov models (27%), decision trees (15%), and combination of decision trees and Markov models (22%) were the most common modelling approaches. Simulation models, including patient-level simulations and discrete event simulations, comprised a further 18% of the models used in the evaluations. The modelling approach varied over the care continuum. Simulation approaches were more common in the prevention and early detection stage, where 34% of models were simulation models. State-transition models, including Markov models, multi-state models, and partitioned survival models, were more common in the treatment and relapsed, refractory or progressive disease stages, where they contributed to 48% of the evaluations applied this model structure, compared with 18% in the prevention and early detection stage.

The majority of evaluations (53%) used a lifetime horizon. The time horizons tended to be slightly longer in the prevention and early detection stage, with 89% of evaluations utilising a lifetime horizon, compared with the treatment phase where 31% of studies applied lifetime horizon. The most commonly used outcome was the QALY, which was applied in 86% of evaluations, followed by LYs (7%).

Information was further extracted on the inclusion of equity, consumer preferences and system dynamics (Table 4 of the ESM). Limitations related to the importance of equity considerations, patient and public preferences, and health system capacity were discussed in 4%, 2% and 13% of articles, respectively. However, they were not formally incorporated into any of the studies identified in this review. The uptake of GM was modelled at the population level in 16 articles (12%) based on assumptions (*n* = 6), the literature (*n* = 9) or clinical claims data (*n* = 1). However, no studies explicitly considered individual preferences for the risks and benefits of genomics and the subsequent impact on consumer behaviour.

## Discussion and Conclusions

Optimal policy decision making in healthcare is a process and not a destination. It requires careful consideration of the decision context, the current state of care, the capacity of the system, and robust health and economics evidence [171]. This systematic review aimed to collate the recent health economics evidence on the cost effectiveness of genomics in cancer control.

Across the cancer care continuum, convergent evidence for the cost effectiveness of GM existed for the prevention and early detection of breast and ovarian cancer, colorectal and endometrial cancers (Lynch syndrome), and multi-cancer testing. There was some evidence for prostate cancer suggesting that using PRS combined with prostate-specific antigen testing may be a cost-effective method for determining which patients require further investigation; however, the evidence was mixed.

The use of GM in the treatment of cancer formed 26% of the articles in this review. The evidence suggested that GM was likely to be cost effective in the treatment of breast and blood cancers. Limited evidence suggested that GM in the treatment of CRC may not be cost effective. These results were difficult to interpret as three of the articles considered the cost effectiveness of treatment (medication or surgery) in specific molecular subgroups rather than modelling a treatment decision based on GM, and one article considered NGS plus artificial intelligence with uncertain results. Of the two articles that considered GM for a treatment decision, GM was demonstrated to be cost effective. For several other cancers, there was insufficient evidence to form high-level conclusions regarding the cost effectiveness of GM for treatment, including gastrointestinal cancers (*n* = 2), gynaecological cancers (*n* = 2), brain and central nervous system cancers (*n* = 2), endocrine cancers (*n* = 1) and tumour agnostic treatments (*n* = 3). For these cancers, additional research may be required.

The management of refractory, relapsed or progressive disease formed the largest part of the review (*n* = 51, 37%). Evidence for the treatment of advanced or metastatic NSCLC showed that the use of GM to identify treatment targets was likely to be cost effective if multigene testing or sequential testing approaches were used. Upfront whole genome sequencing (WGS), or the addition of liquid biopsy to standard testing, was unlikely to be cost effective. For advanced and metastatic RCC, the evidence on cost effectiveness was mixed, with four out of six articles reporting cost-effective results and two reporting borderline or uncertain results. The evidence for advanced and metastatic melanoma showed that GM was unlikely to be cost effective; however, most articles reported the cost effectiveness of GM within a subset or patients with a specific biological marker (BRAF), and therefore the cost effectiveness of using GM to guide treatment decisions is uncertain. As with the treatment phase, there were several cancers for which there was insufficient evidence to infer cost effectiveness, including breast cancer (*n* = 3), cholangiocarcinoma (*n* = 2), leukaemia (*n* = 2), prostate cancer (*n* = 3), gynaecological cancers (*n* = 1), gastrointestinal cancers (*n* = 1) and tumour agnostic treatment (*n* = 1).

While most economic evaluations in this review were conducted in high-income settings, (84%) it is important to acknowledge the transformative potential of GM across jurisdictions. Low- to middle-income countries experience a disproportionate burden of cancer [1, 3], which is further expected to disproportionately increase relative to high-income settings [2], and investment, translational and capacity building efforts in low- to middle-income countries may be slower [172]. More coordinated efforts are needed to support evidence-informed investments in human genomics initiatives globally, and although it is difficult to draw conclusions about the broader transferability of identified economic evaluations across jurisdictions, the insights from this review provide decision makers with evidence to inform GM prioritisation in contexts where value for money evidence converge.

A key challenge in this review was the inference of cost effectiveness, which benchmarks the ICER of the study against an explicit or implicit threshold of decision makers’ willingness to pay for an additional unit of outcome. Quality-adjusted life-years are commonly the recommended outcome in economic evaluations because established thresholds have been identified to support standardised prioritisation decisions across health technologies and clinical contexts and 86% of the studies in the review used QALYs as the primary outcome. In Australia, the commonly used (implicit) threshold used in cost-effectiveness analyses is $50,000 per QALY gained. In the UK, the National Institute for Health and Care Excellence recommends a threshold of UK£20,000–£30,000 per QALY gained, with the National Institute for Health and Care Excellence using a threshold of £100,000 per QALY gained for rare conditions and highly specialised technologies. Given the lack of an explicit threshold of cost effectiveness applicable to GM in cancer control across jurisdictions, studies should identify the probability of cost effectiveness across different decision-making thresholds of cost effectiveness. In this review, 75% of studies conducted probabilistic sensitivity analyses to present this information. Value of information analyses may add further important insights into the implementation of GM.

Although this review provides a high-level summary of the cost-effectiveness evidence, there are broader issues for the implementation of GM requiring consideration, even where cost effectiveness may have been established. For several reasons, including differing levels of access and training data that are built primarily on people with European ancestry, the introduction of GM has the potential to exacerbate pre-existing national and international healthcare inequalities [173, 174]. The review revealed that no evaluations in this space had considered equity within their economic evaluations, and fewer than 5% acknowledged equity as a consideration. Distributional cost-effectiveness analyses may offer important insights to decision makers for the implementation of GM.

Consumers of GM demonstrate a variety of preferences for testing, preventative therapy and treatment [175–184] depending not only on the risks and benefits involved, but also on their personal characteristics, circumstances and experiences. To more accurately estimate the uptake, cost effectiveness, and budget impact of GM and design implementation that optimises the health and economic outcomes of GM, it is necessary to understand consumer preferences, values and priorities.

The introduction of GM, and in particular population screening, has the potential to place a substantial impact on health system capacity and the government budget [185]. While system capacity and constraints were acknowledged in the discussion of 18 of the evaluations, (13%) health economic methods need to advance to explicitly consider the broad system-level implications of GM to safeguard the fiscal sustainability of health systems [186].

This review focused on the cost effectiveness of GM throughout the cancer care continuum. The size and breadth of the review allowed for a comprehensive understanding of the current state of the health economics literature for GM and cancer. The cost-effectiveness evidence can aid decision makers to better understand the types of interventions that are likely to be cost effective and the point on the cancer continuum at which value may be maximised.

While the breadth of this review was a strength, it meant that there was limited scope to discuss nuances of individual papers that may influence results. Findings from each of the studies are highly context dependent. Results are specific to aspects of population, existing care, comparators selected and local decision-making thresholds per unit of outcome achieved. Because of the substantial number of articles identified in this review, and the need for specialty knowledge of every cancer type, a quality assessment was not undertaken; however, indicators of article quality were included in Appendix 1 and Table 4 of the ESM. Systematic reviews can only summarise the available literature, and cost-effectiveness evidence follows the clinical use of GM; therefore, there are areas of developing clinical knowledge that may be high value that were not identified in this review, for example, pancreatic cancer, cancer of unknown primary and rare cancers [187, 188].

To summarise, this systematic review highlighted the points on the cancer care continuum and types of cancer for which GM is likely to be cost effective. Optimising the health and economic outcomes of GM is a continuous process of building capacity and overcoming barriers to ensure an equitable and sustainable translation into clinical practice [15] Investments in human genomics are likely to offer increasing marginal returns, not only by enabling more people to benefit from a genomic diagnosis and targeted therapy, but also by the positive macroeconomic effects of increased employment and tax revenues. [189]. This review will support policy frameworks to incorporate genomics into cancer control. Finally, results from this review highlight that research should aim to expand the evidence base on the cost effectiveness of GM in certain aspects of cancer control and international jurisdictions, broadening the relevance of health economics methods to inform the equitable and sustainable translation of GM.

## Supplementary Information

Below is the link to the electronic supplementary material.

Supplementary file1 (DOCX 81 KB)

Supplementary file2 (XLSX 109 KB)

## Appendix 1: Summary of individual economic evaluations

**Table:**

Article	Population	Intervention	Comparator	ICER^a^	Health outcome	Threshold^a^	Original currency and year	**COI rating**
**Prevention and early detection**								
**Breast and ovarian cancer**								
Manchanda (2018) US [42]	Women ≥ 30 years	FH-based or population-based panel screening	Current family history-based screening	$54,770	QALY	$100,000	USD 2014	2
Manchanda (2018) UK [42]				Dominant	QALY	$46,759	GBP 2014	2
Manchanda (2020) UK [40]	Women ≥30 years	Population-based testing	Family history-based screening	$21,191	QALY	$42,656	USD 2016	3
Manchanda (2020) US [40]				$16,552	QALY	$57,589	USD 2016	3
Manchanda (2020) Netherlands [40]				$25,215	QALY	$50,539	USD 2016	3
Manchanda (2020) China [40]				$23,485	QALY	$15,539	USD 2016	3
Manchanda (2020) Brazil [40]				$20,995	QALY	$15,182	USD 2016	3
Manchanda (2020) India [40]				$31,217	QALY	$6574	USD 2016	3
Pashayan (2018) UK [35]	General population women aged 50-85	(2) Age-based screening (3) Risk-stratified screening	No screening	$981	QALY	$26,000	GBP 2018*	1
Guzauskas (2020) US [36]	General population women ≥30 or ≥45	Population screening	No population screening	$92,600	QALY	$100,000	USD 2019	1
Michaan (2021) Israel [38]	General population women ≥30	Population screening	No screening	$25,354	QALY	$105,780	ISL 2020	2
Oh (2023) US [39]	General population women ≥40	Knowledge of one's BRCA status by age 40	No knowledge of BRCA status	$70,476	QALY	$150,000	USD 2020	3
Michaelson-Cohen (2022) Israel [41]	Ashkenazi Jewish women 30–65	Population screening	(1) High-risk screening (2) Cascade testing	$42,261	QALY	$50,000	USD 2019	1
Patel (2018) UK [44]	Sephardi Jewish women ≥30	Population screening	Family history-based screening	Dominant	QALY	$29,527	GBP 2015	2
Patel (2018) US [44]				$308	QALY	$100,000	USD 2015	2
Muller (2019) Germany [37]	High-risk women ≥35	Targeted screening	No screening	$19,109	QALY	$33,681	EUR 2019*	1
Simoes Correa-Galdani (2021) Brazil [43]	Women ≥30 with FDR or SDR with BRCA cancer	Targeted screening	No screening	$6037	QALY	$6220	BRL 2019	1
Mital (2022) US [45]	General population women 40-49.	8 screen and treat scenarios	No screening	$26,650	QALY	$100,000	USD 2022	1
Wong (2021) Sinagapore [34]	General population women 35 to 74	PRS based stratified surveillance	Standard mammography	Dominant	QALY	**	SGC 2021*	1
Tuffaha (2018) Australia [52]	High-risk women	Proband-based screening	No BRCA testing for probands	$10,730	QALY	$36,008	AUD 2016	1
Sun (2022) China [51]	Women diagnosed with breast cancer	(1) Proband-based screening for all (2) Proband-based screening for high risk	No genetic testing	$8340	QALY	$10,260	USD 2019	2
Hoskins (2019) Canada [46]	Women with epithelial ovarian cancer	Proband-based screening	No genetic testing	$6537	QALY	$36,645	CAD 2018	3
Kemp (2018) UK [54]	Adult women with breast cancer	Risk-based proband screening	No testing	$1225	QALY	$26,184	USD 2018*	3
Ramos (2018) Brazil [47]	Women with breast or ovarian cancer	Proband-based screening	No genetic testing	$908	cancer case avoided	$7543	BRL 2014	4
Kwon (2019) Canada [49]	Women ≥40, FDRs of probands	(2) Population screening (3) risk-reducing bilateral salpingo-oophorectomy for all diagnosed	No testing	$5722	QALY	$73,290	CAD 2018	2
Moya-Alarcon (2019) Spain [48]	Women with ovarian cancer	Proband-based screening	No-testing	$39,941	QALY	$41,995	EUR 2017	3
Hurry (2020) Canada [50]	Women with breast or ovarian cancer	Proband-based screening	No testing	$10,790	QALY	$36,107	CAD 2015	1
Lourencao (2022) Brazil [53]	Women ≥ 30, FDRs or SDRs of probands.	Proband-based screening	No testing	$2157	QALY	$4531	BRL 2021	1
**CRC**								
Thomas (2021) UK [55]	General population ≥30	7 PRS based risk-stratified surveillance strategies	Standard surveillance	Dominant	QALY	$27,072	GBP 2021	1
Cenin (2020) Australia [56]	General population ≥40	PRS-based surveillance	Standard surveillance	$24,225	QALY	$36,008	AUD 2016	2
Van den Puttelar (2023) US [58]	General population	Several risk-stratified surveillance strategies were considered	(1) Uniform screening (2) No screening	$8169	QALY	$100,000	USD 2017	1
Pereira (2019) Portugal; 5 x risk [60]	High-risk population ≥ 40	Risk-stratified surveillance	No genetic testing	$34,623	QALY	$50,376	EUR 2018	1
Naber (2020) US; vs no screening [61]	General population ≥40	PRS-based surveillance	Uniform CRC screening	$19,593	QALY	$69,000	USD 2014	1
Ramdzan (2021) Malaysia [59]	General population ≥18	Genomic testing guided surveillance	Standard surveillance	$196	QALY	**	USD 2019	1
Biltaji (2021) US [57]	General population	Genotype-guided aspirin.	No genetic testing.	$66,243	QALY	$100,000	USD 2019	1
Kang (2020) Australia [62]	FDRs and SDRs of probands	8 different sequential testing strategies (Table S4)	No testing	$22,595	QALY	$39,071	AUD 2017	3
Pastorino (2020) Italy [63]	Adult diagnosed CRC & FDRs	3 different sequential testing strategies (Table S4)	See intervention	$2058	QALY	$36,684	EUR 2020*	1
Azardoost (2021) Iran [64]	Adult diagnosed CRC & FDRs	5 different sequential testing strategies (Table S4)	No screening	$4606	QALY	**	USD 2018	1
Snowsill (2019) UK [65]	Women diagnosed with Endometrial cancer	2 different sequential testing strategies (Table S4)	No testing	$19,221	QALY	$27,072	GBP 2016/17	2
Stinton (2022) UK [67]	Women diagnosed with endometrial cancer	13 different sequential testing strategies (Table S4)	No testing	$12,751	QALY	$27,072	GBP 2016/17	1
Snowsill (2020) UK [66]	Women with endometrial cancer and FDRs/SDRs	4 different sequential testing strategies (Table S4)	No testing	Dominant	QALY	.	GBP 2016/17	1
Salikhanov (2022) [68]	Individuals newly diagnosed CRC and FDRs/SDRs	Genomic screening for all	Standard sequential testing apprroach	$73,571	QALY	$113,085	CHF 2020	1
**Prostate cancer**								
Karlsson (2021) Sweden [69]	General population men 55-59	PRS in addition to PSA	Standard PSA testing	$6736	QALY	$9318	EUR 2019	2
Hao (2022) Sweden [70]	General population men 55-69	PRS in addition to PSA	Standard PSA testing	$43,666	QALY	$53,012	EUR 2019	2
Callender (2019) UK [71]	General population men 55-69.	PRS-based surveillance	No screening Aged-based screening	$21,924	QALY	$36,996	GBP 2016	1
Hendrix (2021) USA [72]	General population men	Several aged-based risk-stratification approaches (Table S4)	Universal screening strategies	$58,400	QALY	$100,000	USD 2018	2
**Multi cancer**								
Taffazoli (2022) USA [73]	General population 50-79	Annual multi-cancer early detection (MCED) testing for 19 solid cancers.	Standard surveillance	Dominant	QALY	$100,000	USD 2021	3
Hackshaw (2021) UK [74]	General population 50-79	Standard surveillance plus MCED	Standard surveillance	Dominant	additional cancer case detected	**	GBP 2019	3
Hackshaw (2021) USA [74]				$7060	additional cancer case detected	**	USD 2019	3
Davidson (2023) Australia [75]	High-risk cancer patients.	(2) Targeted WGS (3) Universal WGS	Targeted gene/gene panel testing	$6740	actionable variant detected	**	AUD 2020	2
Lacaze (2023) Australia [76]	General population	Combined genomic screening for the three high-risk conditions	Clinical criteria-based testing	$16,136	QALY	$34,066	AUD 2022	2
Yeh (2021) USA [77]	General population newborns	Targeted next generation sequencing	Usual care	$244,860	LY	$100,000	USD 2018	2
**Diagnosis, staging and planning**								
Lim (2018) Malaysia [78]	Breast cancer patients ≥40	BRCA mutation testing	Routine Clinical Surveillance (RCS)	$2275	QALY	$9500	USD 2016	1
Hao (2021) USA (direct germline testing vs IHC protocol) [79]	Patients with CRC	8 different testing strategies (Table S4)	See intervention	$11,283	additional case identified	**	USD 2018	2
Balentine (2018) USA [80]	A 40-year-old female with a 1 cm indeterminate thyroid nodule classified as Bethesda III or IV	Genetic testing, positive cases received total thyroidectomy, negative cases received annul ultrasound	Lobectomy and biopsy, positive received total thyroid removal, negative received post-operative care	Dominated	QALY	$100,000	USD 2016	4
Hornberger (2018) USA [81]	Patients with melanoma	Gene expression test	Visual assessment followed by biopsy and histopathologic assessment.	Dominant	Cases identified	**	USD 2017	3
**Treatment**								
**Breast cancer**								
Jongbloed (2023) Netherlands [82]	Patients with breast cancer	(2) abemaciclib for all (3) Minimal residual disease guided abemaciclib	Standard treatment	Dominant	QALY	$56,883	EUR 2021	2
Perez Ramirez (2020) Spain [83]	Patients with breast cancer	Gene expression assay guided treatment	Common prognostic factor guided treatment	Dominant	QALY	$36,684	EUR 2020*	3
Rojas (2023) Colombia [84]	Patients with breast cancer	(1) Oncotype DX guided treatment (2) Mammaprint guided treatment	Chemotherapy for all	Dominant	QALY	$3281	USD 2021	1
Curtit (2023) France [85]	Patients with breast cancer	Oncotype DX guided treatment	Standard of care	Dominant	QALY	$21,465	EUR 2022	3
Ibarrondo (2020) Basque [87]	Patients with breast cancer	Oncotype DX guided treatment	Standard of care	$21,135	QALY	$30,276	EUR 2014	1
Ozmen (2019) Turkey [88]	Patients with breast cancer	Oncotype Dx guided treatment	Standard of care	$7208	QALY	**	USD 2016	1
Wei (2020) China [89]	Patients with breast cancer	CYP2D6*10 guided tamoxifen	Tamoxifen for all	$5016	QALY	$26,508	USD 2017	1
Wei (2020) China [90]	Patients with breast cancer	CYP2D6*10 guided tamoxifen of toremifene	(2) Tamoxifen for all (3) Toremifene for all	$5056	QALY	$26,508	USD 2017	1
Trentham-Dietz (2018) USA [91]	Patients with breast cancer	Hypothetical perfect prognostic test for Non-progressive Ductal carcinoma in situ.	No testing	Dominant	QALY	**	USD 2015	2
Wang (2019) USA, full population [92]	Patients with breast cancer	4 different testing strategies (Table S4)	See intervention	$62,000	QALY	$100,000	USD 2015	3
Wang (2019) USA, low risk [92]				$124,600	QALY	$100,000	USD 2015	3
Wang (2019) USA, high risk [92]				$15,700	QALY	$100,000	USD 2015	3
De Jongh (2022) Netherlands [93]	Patients with breast cancer	(1) Oncotype Dx guided treatment (2) Mammaprint guided treatment	No genomic testing	Dominant	Adverse events	**	EUR 2019	3
Sussel (2022) USA [86]	Patients with breast cancer	7 different testing strategies (Table S4)	See intervention	Dominant	QALY	$100,000	USD 2021	3
**Blood Cancer**								
Alrawashdh (2022) USA [94]	Patients with chronic lymphocytic leukemia	10 interventions were compared (Table S4)	See intervention	Dominant	QALY	$100,000	USD 2020	3
Chatterjee (2023) Canada [95]	Patients with chronic lymphocytic leukemia	Venetoclax combined with obinutuzumab	Patient group specific treatment (Table S4)	Dominant	QALY	$39,203	CAD 2020	3
Slot (2023) Denmark [96]	Patients with chronic lymphocytic leukemia	Venetoclax + obinutuzumab (VenO)	Chlorambucil + obinutuzumab (ClbO).	$10,525	QALY	$25,328	EUR 2022	3
Munir (2023) USA [97]	Patients with chronic lymphocytic leukemia	Acalabrutinib + obinutuzumab	Chlorambucil + obinutuzumab.	$81,960	QALY	$100,000	USD 2021	3
Wei (2022) China [98]	Children with acute lymphoblastic leukemia	NUDT15 testing guided treatment	No genetic testing	Dominant	QALY	$26,508	USD 2017	1
Chen (2018) USA [99]	Patients with diffuse large B-cell lymphoma	Genetic testing guided treatment	Standard RCHOP for all patients	$15,015	QALY	$50,000	USD 2016	4
Regier (2022) Canada [100]	Patients with diffuse large B cell lymphoma	Genetic testing guided treatment	Standard of care	$57,024	QALY	$36,645	CAD 2018	3
Gaultney (2018) Netherlands [101]	Patients with multiple myeloma	Genetic testing guided treatment	Standard of care	Dominant	QALY	**	EUR 2017	3
Gaultney (2018) Germany [101]				Dominant	QALY	**	EUR 2017	3
Gaultney (2018) UK [101]				Dominant	QALY	**	GBP 2017	3
Gaultney (2018) France [101]				Dominant	QALY	**	EUR 2017	3
Gaultney (2018) Spain [101]				Dominant	QALY	**	EUR 2017	3
**CRC**								
Chaudhari (2022) USA [102]	Patients with CRC	(1) 12-gene assay (2) 18-gene assay (3) 482-gene assay	See intervention	$6037	QALY	$50,000	USD 2014	1
Fragoulakis (2023) Italy [103]	Patients with CRC	Molecular testing guided treatment	Standard of care	$16,408	QALY	$61,141	EUR 2020	1
Harty (2018) UK, RAS wt [104]	This study considered three populations of patients with CRC (Table S4)	Cetuximab + Folfori	Folfori	$56,381	QALY	$63,801	GBP 2018*	3
Harty (2018) UK, standard cohort [104]				$167,068	QALY	$63,801	GBP 2018*	3
Lee (2021) Hong Kong, pan-RAS wt left-sided tumour [105]	This study considered three populations of patients with CRC (Table S4)	Chemotherapy plus anti-EGFR therapy	Chemotherapy plus bevicazumab	$76,537	QALY	$97,832	USD 2021*	2
Lee (2021) Hong Kong, KRAS wt [105]				$106,847	QALY	$97,832	USD 2021*	2
Jang (2020) USA [106]	This study considered three populations of patients with CRC (Table S4)	(1) Endoscopic therapy (ET) (2) Laparoscopic colectomy (LC) (3) Open colectomy (OC)	See intervention	$178,765	QALY	$100,000	USD 2019	1
Kacew (2021) USA [107]	Patients with CRC	8 interventions were compared (Table S4)	See intervention	$3333,333	Additional 1% on targeted therapy	**	USD 2021*	1
**Gastrointestinal cancers**								
Liu (2022) China, sintilimab plus a bevacizumab biosimilar [108]	Patients with hepatocellular carcinoma	19 interventions were compared (Table S4)	Sorafenib	$34,959	QALY	$37,653	USD 2022*	1
Liu (2022) China, camrelizumab plus rivoceranib [108]				$22,848	QALY	$37,653	USD 2022*	1
Krepline (2021) USA [109]	Patients with pancreatic cancer	Universal molecular testing strategy.	Selective testing strategy	$121,942	LY	$100,000	USD 2019	2
**Gynaecological cancers**								
Liu (2022) USA [110]	Patients with endometrial cancer.	Lenvatinib plus pembrolizumab	Standard of care	$110,401	QALY	$150,000	USD 2021	1
Orellana (2023) USA, vs no testing [111]	Patients with endometrial cancer.	molecular testing guided treatment	No testing plus 4 other comparators (Table S4)	Dominant	QALY	$100,000	USD 2023*	2
Orellana (2023) USA, vs MMT IHC [111]				$182,797	QALY	$100,000	USD 2023*	2
**Brain and central nervous system cancers**								
Rios (2022) Canada [112]	Children with low-grade glioma.	Molecular testing guided treatment	No molecular testing	Dominant	QALY	**	CAD 2018	1
Ranjan (2023) USA [113]	Patients with glioblastoma multiforme (GBM).	Molecular testing guided therapy	Standard of care	$150,895	LY	$100,000	USD 2023*	3
**Endocrine cancers**								
Tessler (2023) USA [114]	80% female patients diagnosed with low risk differentiated thyroid cancer	Genetic testing guided hemithyroidectomy or total thyroidectomy	Hemithyroidectomy for all	$190	QALY	$50,000	USD 2023*	1
**Tumour agnostic treatments**								
Huygens (2023) Netherlands [115]	59% female cohort of patients with solid tumours	The intervention comprised NTRK gene fusion testing entrectinib in NTRK1 patients and Standard of care in NTRK2 patients.	Standard of care	$207,825	QALY	$97,824	EUR 2020	2
Fragoulakis (2019) Italy [116]	Patients with a histological diagnosis of cancer	DPYD genetic testing guided treatment	Standard of care	Dominant	QALY	**	EUR 2019*	1
Weymann (2022) Canada [117]	Patients with histologically confirmed cancer	Personalised oncogenomics guided treatment	Standard of care	Dominated	LY	$72,216	CAD 2015	3
**Refractory, relapsed or progressive disease**								
**NSCLC**								
Lu (2018) China [122]	Patients with IIIB or IV NSCLC	NGS guided Crizotinib	pemetrexed plus cisplat	$14,384	QALY	$32,000	USD 2016	3
Aguiar Junior (2019) Brazil Erlotnib [123]	Patients with advanced or metastatic NSCLC	erlotin, gefitinib and afatanib	Standard of care chemotherapy	$19,987	QALY	**	BRL 2019*	1
Aguiar Junior (2019) Brazil Gefitinib [123]				$7716	QALY	**	BRL 2019*	1
Aguiar Junior (2019) Brazil Afatinib [123]				$8267	QALY	**	BRL 2019*	1
You (2021) Hong Kong gefitinib [120]	Patients with stage IIIB/IV NSCLC	EGFR testing-guided use of afatinib, erlotinib, gefitinib.	Standard of care chemotherapy	$428,208	QALY	$143,436	USD 2020	1
You (2021) Hong Kong afatinib [120]				$70,690	QALY	$143,436	USD 2020	1
Nadal (2021) Spain [118]	Patients with advanced or metastatic NSCLC	ALK rearrangement testing	No testing	$14,747	QALY	**	EUR 2019	3
Majem (2022) Spain [119]	Patients with advanced NSCLC	ALK testing guided alectinib	Standard of care chemotherapy and/or immunotherapy	$1	NMB	$30,570	EUR 2020	3
Rojo (2022) Spain [121]	Patients with advanced or metastatic NSCLC	ROS1 and sequential testing	No testing ROS1	$21,062	QALY	**	EUR 2021	3
Tan (2020) Singapore [129]	Patients with non-squamous NSCLC.	NGS only testing Sequential (Singleplex) Sequential (Multiplex)	Standard of care	$192	% of patietns on appropriate treatment	**	SGD 2018	3
Zou (2022) USA [130]	Patients with advanced or metastatic non-squamous NSCLC	NGS testing (Table S4)	Single-gene testing (Table S4)	$238,876	QALY	$150,000	USD 2020	3
Arriola (2023) Spain [126]	Patients with advanced or metastatic NSCLC	NGS	Single-gene testing	$27,791	QALY	$32,197	EUR 2022	3
de Alava (2022) Spain [125]	Patients with advanced or metastatic NSCLC	NGS	Sequential single testing	$9749	QALY	$32,197	EUR 2022	3
Wolff (2022) Netherlands [124]	Patients with stage IV NSCLC	NGS/multigene testing	Sequential testing (Table S4)	$74,712	QALY	$85,858	EUR 2022*	3
Steuten (2019) USA [127]	Patients with advanced NSCLC	Multigene testing	Single gene testing	$148,478	LY	$150,000	USD 2017	3
Harvey (2021) USA [128]	Patients with advanced NSCLC in a hypothetical health plan.	30% Comprehensive genomic profiling (CGP) testing and 70% non-CGP testing.	20% CGP testing and 80% testing with a mix of other conventional single-gene assay and non-CGP testing.	$66	LY	**	USD 2018	3
Loubiere (2018) France [132]	Patients with advanced NSCLC.	(2) At least one biomarker testing (3) at least KRAS testing	No biomarker testing approach	$14,474	LY	$54,333	EUR 2015	3
Hofmarcher (2023) USA [131]	Adults patients with advanced NSCLC	Multigene testing	No testing Sequential testing	$94,346	LY	**	USD 2021	3
Hofmarcher (2023) Brazil [131]				Dominant	LY	**	USD 2022	3
Hofmarcher (2023) Germany [131]				Dominant	LY	**	USD 2023	3
Hofmarcher (2023) Poland [131]				Dominant	LY	**	USD 2024	3
Hofmarcher (2023) Turkey [131]				Dominant	LY	**	USD 2025	3
Hofmarcher (2023) South Africa [131]				Dominant	LY	**	USD 2026	3
Hofmarcher (2023) China [131]				Dominant	LY	**	USD 2027	3
Hofmarcher (2023) Japan				Dominant	LY	**	USD 2028	3
Hofmarcher (2023) Australia [131]				Dominant	LY	**	USD 2029	3
Simons (2021) Netherlands [133]	Patients with Inoperable Stage IIIB, C/IV, Non-squamous NSCLC	(2) WGS as a diagnostic test (3) SoC followed by WGS	(1) Standard of care testing	Dominated	QALY	$97,825	EUR 2020	2
Simons (2022) Netherlands [133]	Patients with locally advanced or metastatic NSCLC.	WGS as a diagnostic test.	Standard of care testing	$778,419	QALY	$97,825	EUR 2020	2
Ezeife (2022) Canada [135]	Patients with locally advanced or metastatic NSCLC.	Liquid biopsy plus tissue biopsy	Tissue biopsy alone	Dominant	QALY	**	CAD 2022	1
Englemeier (2023) Germany [136]	Patients with metastatic non-squamous NSCLC	Liquid biopsy plus tissue biopsy	Tissue biopsy only	$65,919	QALY	$36,684	EUR 2020	3
Cho (2023) Korea, first-line [137]	Patients with IIIB or IV NSCLC	(1) Tissue first strategy (2) Plasma-first strategy	Tissue only strategy	Dominant	% patients on appropriate treatment	**	KRW 2023*	1
Cho (2023) Korea, second-line [137]				$10,477	%patients on appropriate treatment	**	KRW2023*	
Schluckbier (2020) Brazil [138]	Patients with adenocarcinoma of NSCLC stage IV	NGS multigene testing	(1) EGFR and ALK testing. (2) EGFR, ALK and ROS1 testing	$165,566	QALY	$150,000	USD 2017	3
Dong (2022) USA [139]	Patients with metastatic adenocarcinoma of NSCLC	(1) Comprehensive genomic profiling (2) Targeted gene panel testing	no tumour profiling strategy	$445,545	QALY	$150,000	USD 2019	2
Leung (2022) Taiwan, vs pembrolizumab [140]	Patients with patients with NSCLC that had experienced disease progression after a platinum-containing systemic therapy	Pembrolizumab Nivolumab Atezolizumab	Docetaxel	$13,896	QALY	$74,203	TWD 2019 (IMF)	1
Leung (2022) Taiwan, vs nivolumab [140]				$52,529	QALY	$74,203	TWD 2019 (IMF)	1
Leung (2022) Taiwan, vs atezolizumab [140]				$52,781	QALY	$74,203	TWD 2019 (IMF)	1
**Breast cancer**								
Saito (2019) Japan [141]	Patients metastatic breast cancer who progressed on chemotherapy	BRCA1/2 mutation profiling	Standard of care chemotherapy	$133,777	QALY	$91,146	JPY 2018	1
Ren (2023) China [142]	Patients with metastatic breast cancer	Neratinib plus capecitabine (N + C)	lapatinib plus capecitabine (L + C)	Dominant	QALY	$36,000	USD 2022	1
Pennarun (2022) Taiwan [143]	Patients with invasive breast cancer who underwent surgery as their primary treatment	RI-DR® 18-gene prognostic assay	No RI-DR gene testing	$5675	QALY	$25,787	TWD 2022* (IMF)	3
**RCC**								
Nazha (2018) Canada [144]	Patients with metastatic RCC.	Sunitinib	Pazopanib	$53,469	QALY	$79,524	CAD 2017	1
Zhu (2023) USA [145]	Patients with advanced RCC	(1) LP group: lenvatinib plus pembrolizumab (2) LE group: lenvatinib plus everolimus	(3) sunitinib group	$131,656	QALY	$150,000	USD 2021	1
Chen (2022) USA [146]	Patients with metastatic RCC.	Pharmacokinetic guided sunitinib.	Standard dose of sunitinib.	Dominant	QALY	$100,000	USD 2020	1
Chen (2022) China [146]				Dominant	QALY	$31,500	USD 2020	1
Redig (2019) Sweden, period 1 [147]	Patients with metastatic RCC	Targeted therapies: imatinib, gefitinib, erlotinib, sunitinib, sorafenib, dasatinib, lapatinib, nilotinib, everolimus, pazopanib, and axitinib	Therapies used pre-TT introduction	$78,656	LY	$84,000	USD 2014	3
Redig (2019) Sweden, period 2 [147]				$34,132	LY	$84,000	USD 2014	3
Chandler (2023) Japan, vs nivolumab [148]	Patients with advanced RCC	Cabozantinib	Everolimus Axitinib Nivolumab	Dominant	QALY	$57,251	JPY 2023*	3
Chandler (2023) Japan, vs everorlimus [148]				$41,034	QALY	$57,251	JPY 2023*	3
Chandler (2023) Japan, vs axitinib [148]				$16,970	QALY	$57,251	JPY 2023*	3
Meng (2018) England, vs nivolumab [149]	Patients with advanced RCC	Cabozantinib	Everolimus Axitinib Nivolumab	Dominant	QALY	$127,602	GBP 2017	1
Meng (2018) England, vs axitinib [149]				$126,284	QALY	$127,602	GBP 2017	1
Meng (2018) England, vs everorlimus [149]				$175,389	QALY	$127,602	GBP 2017	1
**Melanoma**								
Mojtahed (2021) USA [152]	Patients with Stage III BRAF V600 E mutated melanoma.	Five treatment arms (Table S4)	No adjuvant treatment	$95,758	QALY	$100,000	USD 2019	2
Wahler (2022) Germany [156]	Patients with resected BRAF V600 mutant stage III melanoma.	(1) Dabrafenib and trametinib (2) Nivolumab irrespective of BRAF-mutation status	Observation	$40,568	QALY	**	EUR 2022*	2
Mulder (2021) Netherlands [159]	Patients with high-risk stage III melanoma.	(1) Immune checkpoint inhibitors (2) Targeted therapy	Routine surveillance (Placebo)	$45,840	QALY	$61,140	EUR 2020	2
Charpentier (2023) France [158]	Patients with unresectable and metastatic melanoma (2 populations Table S4)	Targeted therapy, immunotherapy	Palliative care, chemotherapy, immunotherapy	$110,278	LY	$122,281	EUR 2020	1
Bensimon (2020) USA [150]	Patients with complete resection of high-risk stage IIIA, IIIB, or IIIC melanoma]	Pembrolizumab	Targeted therapy Dabrafenib + trametinib	Dominated	QALY	$100,000	USD 2019	3
Wu (2020) USA [151]	Patients with advanced melanoma	4 BRAF test and treat strategies (Table S4).	4 immunotherapy strategies (Table S4)	Dominated	QALY	$150,000	USD 2019	1
Li (2023) USA [154]	Patients with BRAF mutation advanced melanoma.	4 treatment strategies (Table S4)	See intervention	$319,113	QALY	$150,000	USD 2016	1
Gil-Rojas (2021) Colombia (Adjusted in SR) [153]	Patients with unresectable or metastatic melanoma with the BRAFV600 mutation.	(D+T) dabafenib in combination with trametinib	9 comparators (Table S4)	$691,829	QALY	$17,393	COP 2018	3
Tarhini (2019) USA [157]	Patients with advanced BRAF-mutant melanoma	3 strategies combining anti-PD-1, BRAF + MEK inhibitors (Table S4)	See intervention	$79,124	QALY	$150,000	USD 2016	3
Kandel (2022) France [155]	Patients with advanced primary melanoma	Five sequences for the first and subsequent line of treatment of the BRAF-m and BRAF wt populations (Table S4)	See intervention	Dominant	QALY	**	EUR 2019	2
**Cholangiocarcinoma**								
Chen (2023) Taiwan, vs oxaliplatin, L-folinic acid and fuorouracil [160]	Patients with cholangiocarcinoma who have progressed on at least one therapy	Pemigatinib	(1) Oxaliplatin (2) leucovorin	$189,805	QALY	$95,595	TWD 2023 (IMF)	2
Chen (2023) Taiwan, vs leucovorin and fuorouracil [160]				$175,624	QALY	$95,595	TWD 2023 (IMF)	2
Chueh (2023) Taiwan [161]	Patients with intrahepatic cholangiocarcinoma	Pemigatinib monotherapy and mFOLFOX regimens based on FGFR2 status	Fluorouracil chemotherapy (5-FU)	$123,077	QALY	$104,264	TWD 2021 (IMF)	2
**Leukemia**								
Pandya (2021) USA, vs standard care [162]	Patients with FLT3 mutated acute myeloid leukemia	Gilteritinib	(1) Standard care (2) Best supportive care	$115,192	QALY	$150,000	USD 2019	3
Pandya (2021) USA, vs best supportive care [162]				$107,435	QALY	$150,000	USD 2019	3
**Prostate cancer**								
Parmar (2021) Canada [163]	Patients with metastatic castration-sensitive prostate cancer	apalutamide + ADT	ADT alone	$129,135	QALY	$78,406	CAD 2020	1
Barbier (2022) Switzerland [164]	Patients with metastatic hormone sensitive prostate cancer,	5 treatment strategies were considered (Table S4)	See intervention	$22,293	QALY	$80,091	EUR 2021	1
Su (2020) USA, 3 variants [165]	Patients with metastatic castration-resistant prostate cancer. Comparison was modelled in 2 populations (Table S4)	Olaparib	Standard of care	$116,903	QALY	$150,000	USD 2019	1
Su (2020) USA, 15 variants [165]				Dominant	QALY	$150,000	USD 2019	1
**Gastrointestinal cancers**								
Banerjee (2020) USA [166]	Patients with metastatic gastrointestinal stromal tumours	Molecular testing guided treatment (Table S4)	Empirical imatinib therapy	$92,100	QALY	$100,000	USD 2019	3
**Gynaecological cancers**								
Richardson (2023) USA, chemo + bevacizumab (bev) followed by cemiplimab [167]	Patients with recurrent and metastatic cervical cancer	3 potential treatment sequences (Table S4)	Standard of care	$576,121	QALY	$150,000	USD 2022	3
Richardson (2023) USA, chemo + bev followed by pembrolizumab [167]				$847,361	QALY	$150,000	USD 2022	3
Richardson (2023) USA, chemo + bev + pembro followed by tisotumab [167]				$887,564	QALY	$150,000	USD 2022	3
**Cancer agnostic treatment**								
Vellekoop (2023) England [168]	Patients with locally advanced or metastatic solid tumours	4 NTRK testing strategies using NGS were compared (Table S4)	No testing	$102,683	QALY	$40,955	EUR 2021	1
Vellekoop (2023) Hungary [168]				$98,427	QALY	$15,173	HUF 2021	1
Vellekoop (2023) Netherlands [168]				$186,375	QALY	$91,012	EUR 2021	1
**Palliative care and end-of-life**								
Ree (2022) Norway, RECOURSE [169]	Adult patients with refractory end-stage cancer with a life expectancy of at least 3 months.	Genomic molecularly matched off-label therapies for end-stage cancer	Comparison based on two trials (Table S4)	$154,395	QALY	$68,953	EUR 2020	3
Ree (2022) Norway, CORRECT [169]				$134,012	QALY	$68,953	EUR 2020	3
Edwards (2018) UK [170]	People with previously treated advanced or metastatic RCC who received previous VEGF-targeted therapy	Axitinib, cabozantinib, everolimus, nivolumab and sunitinib	Best supportive care	$66,436	QALY	$73,816	GBP 2015	1

*ALL* acute lymphoblastic leukemia, *CRC* colorectal cancer, *FDR* first-degree relative, *NSCLC* non-small cell lung cancer, *PRS* polygenic risk score, *PSA* prostate specific antigen, *RCC* renal cell carcinoma

*Cost year taken from year of publication as it was not made explicit in the evaluation ** Threshold not explicitly stated in evaluation. Conflict of interest was evaluated by reviewer MB using the following rating system: (1) the manuscript reported on COI and noted that there was no potential COI; (2) the manuscript acknowledged that authors had potential COI, but these authors were not lead or corresponding authors; (3) the lead or corresponding author noted potential COI; 4) the manuscript did not report COI. Where table refers to Table S4 it is indicating that more detail is available in Supplementary Table 4

^a^Values have been converted into USD in the reference year of the study

## Declarations

### Availability of Data and Material

All data used to generate this review are available online from the references provided. Data extracted are presented in the article and the supplementary Appendix.
