# Supplementary material for: The Cost Effectiveness of Genomic Medicine in Cancer Control: A Systematic Literature Review
Source: Appl Health Econ Health Policy. 2025 Mar 29;23(3):359–93. doi: 10.1007/s40258-025-00949-w (PMC12053027; doi:10.1007/s40258-025-00949-w)

Supplementary Tables

Supplementary Table 1: Search terms

Supplementary Table 2: Inclusion/exclusion criteria

Supplementary Table 3: Data extracted during review

Supplementary Table 4: Additional extracted data

Supplementary Table 5: Income distribution countries

Supplementary Figure 1: Geographical distribution of papers

Supplementary Table 1: Search terms

| Concept | Search terms |
| --- | --- |
| Genomics | Whole genome sequenc*, WGS, Whole exome sequenc*, WES, Genetic* testing, Genom* testing, Genetic* sequencing, Genom* sequencing, Exom* testing, Exom* sequencing, Sequence analysis, Genetic profiling, Genom* profiling, Direct to consumer testing, Direct to consumer genetic*, Direct to consumer genom*, Personal genom*, Individual genetic*  Individual genom*, Genetic susceptibility test*, Genomic risk profiling, Genome based testing, massively parallel sequencing, next generation sequencing, high throughput nucleotide sequencing, miseq, hiseq, whole adj2 sequencing, pharmacogenetic*, pharmacogenomic*, (genom* or precision or personal* or stratif* or individuali* or target* or P4) adj1 (medic* or treatment or therap*), genetic counsel*, biomolecular technolog*, polygenic risk score*, PRS, Genomic* risk score*, Genomic score*, GRS, genomic*-informed, multi-cancer early detection test*, MCED,  **MeSH terms:** whole genome sequencing, exome sequencing, genetic testing, sequence analysis, high-throughput nucleotide sequencing, pharmacogenetics |
| Economic evaluation | **Economic evaluation** Economic evaluation, health technology assessment*, HTA, QALY, life year, cost* adj2 benefit*, cost* adj2 utilit*, cost* adj2 effectiv*, cost* adj 2 minimi*, cost* adj2 analy*, cost* adj2 outcome*, cost* adj2 consequence*, cost* adj2 stud*, economic adj2 analy*, resource adj2 allocation, cost*, willingness adj2 pay |
| Cancer | Cancer*, Neoplasm*, Neoplasia*, Tumour*, Tumor* Malignanc*, Carcinoma*  **Mesh terms:** neoplasms |

Supplementary Table 2: Inclusion/exclusion criteria

| Inclusion | Exclusion |
| --- | --- |
| Full economic evaluation (CEA, CUA, CBA, CC)  Relates to any cancer  Relates to any point on the care continuum  Includes the use of genomic screening/genomic testing  Adults or children | Conference abstracts  Review articles  Case reports  Guidelines  Conference statements  Cost-minimisation analysis |

Abbreviations: CBA, cost-benefit analysis; CC, cost-consequence; CEA, cost-effectiveness analysis; CUA, cost-utility analysis

Supplementary Table 3: Data extracted during review

| Data collected |
| --- |
| Type of cancer, point on the cancer continuum, germline or somatic testing, type of health economic evaluation. perspective, country, type of evaluation, model type, model structure cost, outcomes cost-effectiveness estimates, time horizon, intervention, comparator, utility source, willingness-to-pay (WTP) threshold applied, cost source, incremental costs, incremental outcomes, ICER, probability of cost-effectiveness, conclusions, and whether equity, system capacity and patient preferences were included, COI reported, COI information |

Supplementary Table 4: Economic Evaluation Characteristics is available in attached Excel file

Supplementary Table 5: Country income distribution

| Country | Count | Middle or lower income | LMIC | Income |
| --- | --- | --- | --- | --- |
| **United States** | 46 | 0 | 0 | High |
| **United Kingdom** | 14 | 0 | 0 | High |
| **Netherlands** | 10 | 0 | 0 | High |
| **China** | 10 | 0 | 1 | Upper-Middle |
| **Brazil** | 7 | 0 | 1 | Upper-Middle |
| **India** | 1 | 1 | 1 | Lower-Middle |
| **Israel** | 2 | 0 | 0 | High |
| **Germany** | 5 | 0 | 0 | High |
| **Singapore** | 2 | 0 | 0 | High |
| **Australia** | 6 | 0 | 0 | High |
| **Canada** | 10 | 0 | 0 | High |
| **Spain** | 8 | 0 | 0 | High |
| **Portugal** | 1 | 0 | 0 | High |
| **Malaysia** | 2 | 0 | 1 | Upper-Middle |
| **Italy** | 3 | 0 | 0 | High |
| **Iran** | 1 | 0 | 1 | Upper-Middle |
| **Sweden** | 3 | 0 | 0 | High |
| **Colombia** | 2 | 0 | 1 | Upper-Middle |
| **France** | 5 | 0 | 0 | High |
| **Basque** | 1 | 0 | 0 | High |
| **Turkey** | 2 | 0 | 1 | High |
| **Denmark** | 1 | 0 | 0 | High |
| **Hong Kong** | 2 | 0 | 0 | High |
| **Poland** | 1 | 0 | 0 | High |
| **South Africa** | 1 | 0 | 1 | High |
| **Japan** | 3 | 0 | 0 | High |
| **Korea** | 1 | 0 | 0 | High |
| **Taiwan** | 4 | 0 | 0 | High |
| **England** | 2 | 0 | 0 | High |
| **Switzerland** | 1 | 0 | 0 | High |
| **Hungary** | 1 | 0 | 0 | High |
| **Norway** | 1 | 0 | 0 | High |

Supplementary Figure 1: Geographical distribution of evaluations


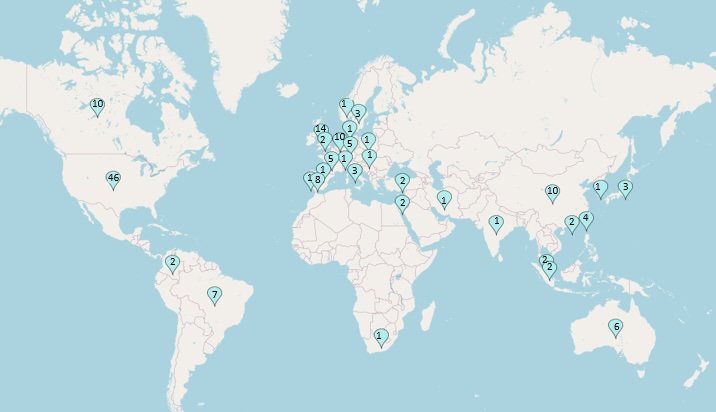

Supplement: Supplementary file 1 — Supplementary file1 (DOCX 81 KB) [file 40258_2025_949_MOESM1_ESM.docx]
